# JAK-STAT and IL-17 pathway dysregulation underlies persistent immune dysfunction in ART-experienced people living with HIV in Ghana

**DOI:** 10.3389/fimmu.2026.1753475

**Published:** 2026-02-10

**Authors:** Mark Appeaning, Edwin Magomere, Nana Ama Yeboaa Amoako, Kirk Elorm Kouffie, Kesego Tapela, Charles Ochieng’ Olwal, Jones Amo Amponsah, Stella Nartey, Rosalynn Baah-Danquah, Salome Tettey Frimpong, Seyram Tetteh Quarshie, Samuel Efa-Quayson, Francis Broni, Felix E. Nenyewodey, James Abugri, Gloria Akosua Ansa, Evelyn Yayra Bonney, Peter Kojo Quashie

**Affiliations:** 1 West African Centre for Cell Biology of Infectious Pathogens (WACCBIP), College of Basic and Applied Sciences, University of Ghana, Accra, Ghana; 2 Department of Biochemistry Cell and Molecular Biology, School of Biological Sciences, College of Basic and Applied Sciences, University of Ghana, Accra, Ghana; 3 Department of Medical Laboratory Science, Faculty of Health and Allied Sciences, Koforidua Technical University, Koforidua, Ghana; 4 Noguchi Memorial Institute for Medical Research, University of Ghana, Accra, Ghana; 5 University of Ghana Health Services, Public Health Department, Accra, Ghana; 6 Fevers Unit, Greater Accra Regional Hospital, Accra, Ghana; 7 HIV Clinic, Ho Municipal Hospital, Ho, Ghana; 8 Upper East Regional Hospital, Bolgatanga, Ghana; 9 Biomedical Science Department, Navrongo Health Research Centre, Navrongo, Ghana; 10 Department of Biochemistry and Forensic Sciences, School of Chemical and Biochemical Sciences, C. K. Tedam University of Technology and Applied Sciences, Navrongo, Ghana; 11 The Francis Crick Institute, London, United Kingdom

**Keywords:** antiretroviral therapy, cytokines and chemokines, HIV, IL-17, immune activation, JAK-STAT, West Africa, WHICH study

## Abstract

**Introduction:**

Chronic immune activation and inflammation are central to HIV pathogenesis and persist despite antiretroviral therapy (ART), contributing to non-AIDS comorbidities. The HIV epidemic in West Africa is distinct, marked by the coexistence of HIV-1, HIV-2 in circulation as well as recombinant forms, yet immune responses in this region remain under-investigated. This study examined how ART modulates cytokine and chemokine signaling in Ghanaian people living with HIV (PLWH), with emphasis on biomarkers of immune dysfunction and treatment response.

**Methods:**

Plasma concentrations of 25 cytokines and chemokines were quantified using Luminex multiplex assays in 247 participants: ART-naïve (n=141), post-ART at 6-months (n=52) and 12-months (n=23), ART-experienced (n=74), and HIV-negative controls (n=32). Differentially expressed cytokines, cytokine network analysis, and pathway enrichment analyses, including Gene Ontology (GO) and Kyoto Encyclopedia of Genes and Genomes (KEGG) were performed using R-anchored packages. Correlations between cytokine levels and viral load were also evaluated. Cox proportional hazards regression was applied to identify biomarker of HIV disease progression and predictive modelling using Least Absolute Shrinkage and Selection Operator (LASSO) regression, Random Forest (RF), and Gradient Boosting Machine (GBM).

**Results:**

ART-naïve individuals exhibited elevated pro-inflammatory (IL-6, IL-12/IL-23p40, IL-2, IL-15, IL-2R), and chemotactic (MCP-1, IP-10, MIG) cytokines, alongside reduced IL-1β and IL-1Ra. ART significantly reduced inflammatory cytokines, but paradoxically increased RANTES and Eotaxin. IL-1Ra emerged as the central node in cytokine interaction networks, while IP-10 positively and RANTES negatively correlated with viral load. Lower IL-1β and IL-10 levels predicted virologic control, whereas elevated GM-CSF was linked to persistent viraemia. Machine learning modelling identified RANTES, IP-10, IL-12/IL-23p40, IL-7, and IL-2R as the strongest predictors of viral load. Pathway enrichment analysis revealed upregulation of chemokine-mediated signaling and eosinophil chemotaxis, but downregulation of leukocyte activation, IL-17, and JAK-STAT signaling.

**Conclusion:**

ART attenuates systemic inflammation and partially restores immune balance in PLWH in Ghana, but recovery remains functionally dysregulated, with persistent chemotactic signaling and impaired mucosal and JAK-STAT–mediated immunity. IL-1β, IL-10, GM-CSF, RANTES, and IP-10 emerge as prognostic markers of disease progression and potential targets for adjunctive immunotherapies. These findings underscore the need for immune-modulatory strategies to optimize ART outcomes in West Africa.

## Introduction

The human immunodeficiency virus (HIV) epidemic in West Africa is uniquely characterized by the co-circulation of different HIV-1 subtypes, circulating recombinant forms (CRFs) predominantly CRF02_AG, unique recombinant forms (URFs) and HIV-2, and (1, 2). Unlike other regions where a single subtype dominates, this viral heterogeneity complicates treatment strategies and epidemiological tracking. HIV-2, in particular, remains endemic in West Africa, where it overlaps with a high burden of co-infections such as tuberculosis and an increasing prevalence of non-communicable diseases—factors that collectively influence treatment response and disease progression (3).

The global scale-up of ART has dramatically improved the prognosis of people living with HIV (PLWH), transforming it into a manageable chronic condition, particularly in high-income countries (HICs) (4). However, the benefits of ART are not equitably distributed (5). In low- and middle-income countries (LMICs), including many in Sub-Saharan Africa, HIV remains associated with high morbidity and mortality. One major contributor to this disparity is unequal access to newer, less toxic, and more effective ART regimens (5–7).

Despite widespread access to antiretroviral therapy (ART), treatment response remains variable. While many individuals achieve viral suppression, persistent immune activation and systemic inflammation are common and contribute to virologic non-suppression and non-AIDS-related comorbidities (8). In Ghana, unusually high rates of viral non-suppression 6–12 months post-ART initiation have been reported, in contrast to findings from DTG-anchored therapy in other regions outside West Africa (9). Interestingly, immune recovery occurred despite persistent viremia, suggesting distinct immune response dynamics and regional differences in treatment efficacy that warrant further investigation. Similar trends in viral non-suppression (VNS) have been documented across sub-Saharan Africa among adolescents and young adults in Tanzania and Kenya, and a recent meta-analysis estimated that two in every ten people living with HIV on ART experience VNS, posing a major challenge to achieving the UNAIDS third 95% target (10–12).

Cytokine and chemokine dysregulation play a central role in HIV pathogenesis. Pro-inflammatory mediators such as TNF-α, IL-1, IL-2, IL-6, IL-12, and GM-CSF enhance viral replication, whereas others, including TGF-β, IL-4, IL-10, IL-13, and IFN-γ, may suppress it (13). Yet, in West Africa and particularly in Ghana, the immunopathogenesis of HIV remains understudied.

Given the region’s complex epidemiology, characterized by diverse viral subtypes, high co-infection burden, and socio-economic disparities, immunological investigations are essential. Therefore, we examine how ART modulates immune responses in people living with HIV (PLWH) in Ghana, focusing on cytokine and chemokine dynamics. We employed machine learning, performed survival analysis, cytokine interaction networks, and pathway enrichment to identify key immune mediators linked to virologic control or persistent replication, providing mechanistic insights and potential biomarkers to understand ART outcomes in the region.

## Materials and methods

### Study design and participant

This work was conducted as part of the West African Centre for Cell Biology of Infectious Pathogens (WACCBIP) Long-term HIV Infection Cohort (WHICH Study) (9). A longitudinal design was employed for ART-naïve (M0) participants—PLWH who were yet to start ART and then followed up at six- and twelve-months post ART. In parallel, a cross-sectional design was used for ART-experienced participants (T_E0) who had received ART for at least six months at enrolment. Recruitment was conducted between July 2022 and September 2024 at Greater Accra Regional Hospital, University of Ghana Hospital–Legon, Tema General Hospital, Ho Municipal Hospital, Upper East Regional Hospital (Bolgatanga), and War Memorial Hospital (Navrongo). Healthy controls (CON) were recruited from the International Maritime Hospital and the West African Centre for Cell Biology of Infectious Pathogens. In total, the study enrolled 32 healthy controls, 141 ART-naïve, 52 at six months, 23 at twelve months, and 74 ART-experienced.

### Sample collection, processing and HIV-1 viral load quantification

Venous blood (10 mL) was collected into BD Vacutainer^®^ K2EDTA and SST™ tubes (BD Biosciences, UK). Plasma and serum were separated by centrifugation (2500 rpm for 10 minutes) and stored at –80°C. Peripheral blood mononuclear cells (PBMCs) and red blood cells (RBCs) were also isolated and cryopreserved.

Plasma viral RNA was extracted using the Quick-RNA Viral Kit (Zymo Research, Cat. No. R1035) following the manufacturer’s protocol. HIV-1 viral load was quantified using the Bosphore^®^ HIV-1 Quantification Kit (Anatolia Geneworks, Cat. No. ABHIQ3) on the QuantStudio™ 5 Real-Time PCR System (Applied Biosystems). Viral load values, initially obtained in International Units/mL (IU/mL), were converted to copies/mL using a conversion factor of 1 IU = 0.7 copies/ml, as specified by the manufacturer, this was to ensure comparability with the WHO International Standard for HIV RNA NAT assays (NBSIC code 97/650).

### Plasma cytokine and chemokine measurement

Plasma concentration of various cytokines and chemokines were evaluated using the Human Cytokine Magnetic 25-Plex Panel (Invitrogen, Thermo Fisher Scientific, USA). The cytokines assessed were granulocyte-macrophage colony-stimulating factor (GM-CSF), interferon alpha (IFN-α), interferon beta (IFN-β), interleukin-1 receptor antagonist (IL-1Ra), IL-1 beta (IL-1β), IL-2, IL-2 receptor (IL-2R), IL-4, IL-5, IL-6, IL-8 (CXCL8), IL-10, IL-12/IL-23p40, IL-13, IL-15, IL-17A, and tumor necrosis factor alpha (TNF-α). The chemokines were regulated on activation, normal T cell expressed and secreted (RANTES/CCL5), macrophage inflammatory protein-1 alpha (MIP-1α/CCL3), Eotaxin (CCL11), macrophage inflammatory protein-1 beta (MIP-1β/CCL4), monocyte chemoattractant protein-1 (MCP-1/CCL2), monokine induced by gamma interferon (MIG/CXCL9) and interferon gamma-induced protein 10 (IP-10/CXCL10).

The assay was conducted following the manufacturer’s instructions and as previously reported by Tapela et al. (14). Briefly, in a 96-well plate, 25 µL of antibody-coated beads were added and washed. Then, 100 µL of samples, standards, and blanks were added and incubated for 2 hours with shaking at 250 rpm on a MicroPlate Shaker (Thermo Scientific, Korea). Subsequently, 100 µL of biotinylated detector antibody was added and incubated for 1 hour. Thereafter 100 µL of streptavidin-RPE was added incubated for 30 minutes, wells were washed, and 150 µL of wash buffer was added. The assay was read using a Luminex MAGPIX system (Luminex Corporation, Austin, TX, USA) and data analyzed using xPONENT™ software (v4.3.229), according to the manufacturer’s protocol.

### Cytokines and chemokines as predictors of HIV progression

Cox proportional hazards regression was used to evaluate associations between cytokine levels and HIV progression at baseline, six months, and twelve months. Hazard ratios (HRs) and 95% confidence intervals (CIs) were estimated with the survival package in R and visualized with forest plots generated using the survminer package (15). Model significance was assessed with the log-rank test.

### Modelling cytokines and chemokines as predictors of viral load

To identify cytokine predictors of viral load, we applied three machine learning modelling approaches: Least Absolute Shrinkage and Selection Operator (LASSO) regression to select cytokines with strong linear associations, Random Forest (RF) to capture non-linear interactions and estimate variable importance based on percentage increase in mean squared error (%IncMSE) and Gradient Boosting Machine (GBM) to assess relative influence across sequential decision trees (16, 17). Model performance was evaluated by comparing predicted versus observed log viral load on the test set. Variable importance plots identified top predictors. Partial dependence plots (PDPs) were used to visualize non-linear effects of the top 10 cytokines.

### Network and pathway analysis

Cytokine–protein interaction networks were constructed using the STRING database (STRINGdb) (18); https://string-db.org/, focusing on cytokines differentially expressed between ART-naïve and ART-experienced groups. To ensure high-confidence interactions, only experimentally validated and high-confidence STRING interactions (confidence score > 0.7) were used.

Network structure was analyzed by computing key centrality measures, including degree centrality and betweenness centrality, to identify highly interconnected and functionally influential cytokines. The network was visualized using the igraph package in R (19). Functional enrichment was performed with clusterProfiler (20), using Gene Ontology (GO) (biological process, molecular function, cellular component) and Kyoto Encyclopedia of Genes and Genomes (KEGG) databases (21, 22). Gene names were retrieved from UniProt (23), and terms with false discovery rate (FDR) < 0.05 were considered significant.

### Data processing and statistical analysis

Cytokines and chemokines were categorized into three functional groups based on their established roles in HIV pathogenesis. Pro-inflammatory cytokines were IL-1β, IL-5, IL-6, TNF-α, IL-12/IL-23p40, GM-CSF, IFN-γ, IFN-α, IL-2, IL-7, IL-15, IL-2R, and IL-17. Anti-inflammatory; IL-10, IL-1Ra, IL-4 and IL-13. Chemokines were MIP-1α (CCL3), MIP-1β (CCL4), RANTES, Eotaxin, MCP-1, IL-8, MIG, and IP-10 (24–26). Raw data was processed in Microsoft Excel and analyzed using GraphPad prism software Inc version 8 (GraphPad Software, San Diego, CA, USA) and open resource packages anchored in R software version 4.1.0 (R Development Core Team, Vienna, Austria, and R studio Version 2024.12.0.467). Cytokine and chemokine concentrations were expressed as Net Median Fluorescence Intensity (net MFI). Viral load, cytokine, and chemokine data were log_10_-transformed. Group comparisons were made using the Kruskal–Wallis test with Dunn’s *post hoc* test. Spearman’s correlations assessed associations between cytokines and viral load. Significance was set at p < 0.05.

## Results

### Participant characteristics and HIV-1 viral load dynamics

HIV-1 viral load remained high and unsuppressed among ART-naive their longitudinal follow-up pairs (**Table 1**). In contrast, viral suppression was observed in ART-experienced participants at the time of recruitment, the majority of whom had been on treatment for more than five years.

**Table 1:** Participant characteristics and HIV-1 viral load dynamics.

Participant/Sample Description	Age median (IQR)	Log_10_ viral load median (IQR)	Mean treatment duration
Control (n=32)	27 (23.4 - 31.8)	NA	NA
ART-Naïve/M0 (n= 141)	35(29 - 43)	5.2(4.7 - 5.7)	0
M06 (n=52)		4.6(3.3 - 4.9)	6 months
M12 (n=23)		3.9 (3.4 - 4.8)	12 months
ART-Experienced (n=74)	42 (35 - 51.5)	2.8 (2.5 - 3.0)	> 5 years

NA, not applicable; IQR, Interquartile range; M06, six-month follow up; M12, twelve-month follow up.

### Cytokine alterations in ART-naïve and ART-experienced participants

**Figure 1:**
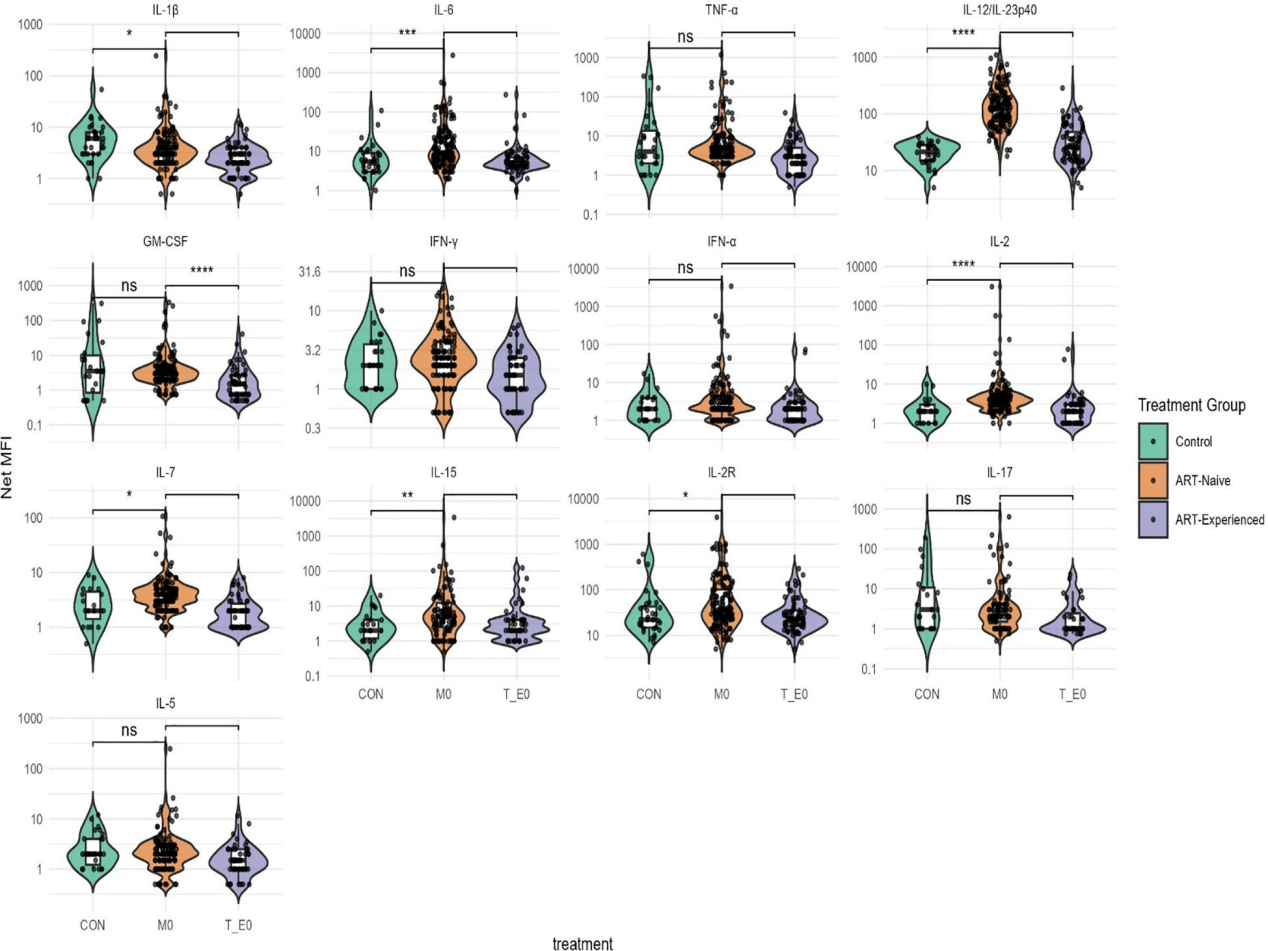
Pro-inflammatory cytokine levels in ART-naïve, ART-experienced, and control. Cytokine concentrations were measured by multiplex immunoassay and are expressed as Net MFI (log scale). The median is shown by the horizontal line within each box, while the lower and upper bounds represent the 25th and 75th percentiles, respectively. Violin plots illustrate the overall distribution of values within each group. Statistical comparisons were performed using Kruskal–Wallis test with Dunn’s *post hoc* correction. Significance thresholds are denoted as follows: ****p < 0.0001; ***p < 0.001; **p < 0.01; *p < 0.05; ns, not significant.

**Figure 2:**
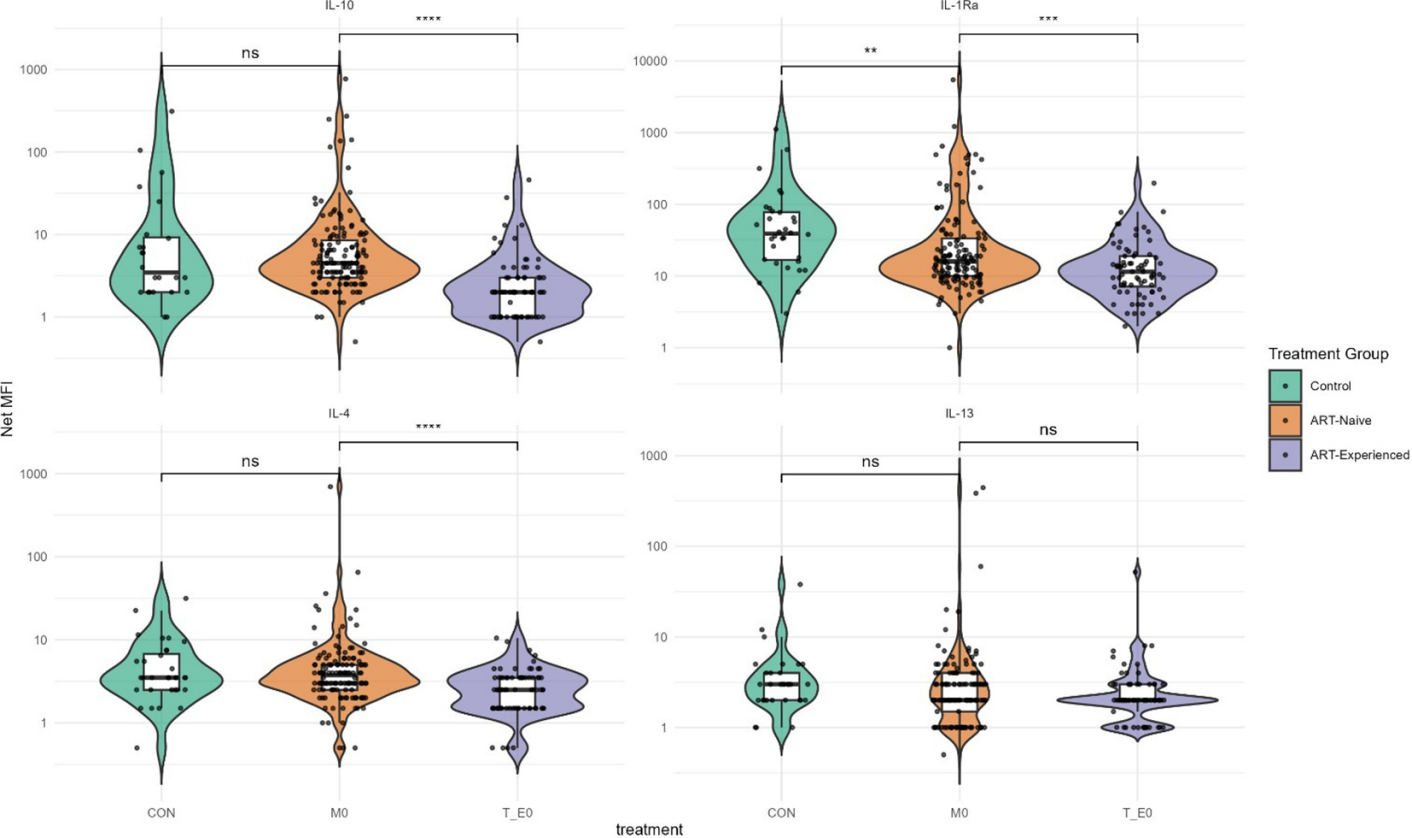
Anti-inflammatory cytokine levels in ART-naïve, ART-experienced, and control. Concentrations of IL-10, IL-1Ra (interleukin-1 receptor antagonist), IL-4, and IL-13 were measured by multiplex immunoassay and expressed as Net MFI (log scale). The median is shown by the horizontal line within each box, with the lower and upper bounds representing the 25th and 75th percentiles, respectively. Violin plots illustrate the overall distribution of values within each group. Statistical comparisons were performed using Kruskal–Wallis test with Dunn’s *post hoc* correction, with significance thresholds indicated as follows: ****p < 0.0001; ***p < 0.001; **p < 0.01; *p < 0.05; ns, not significant.

**Figure 3:**
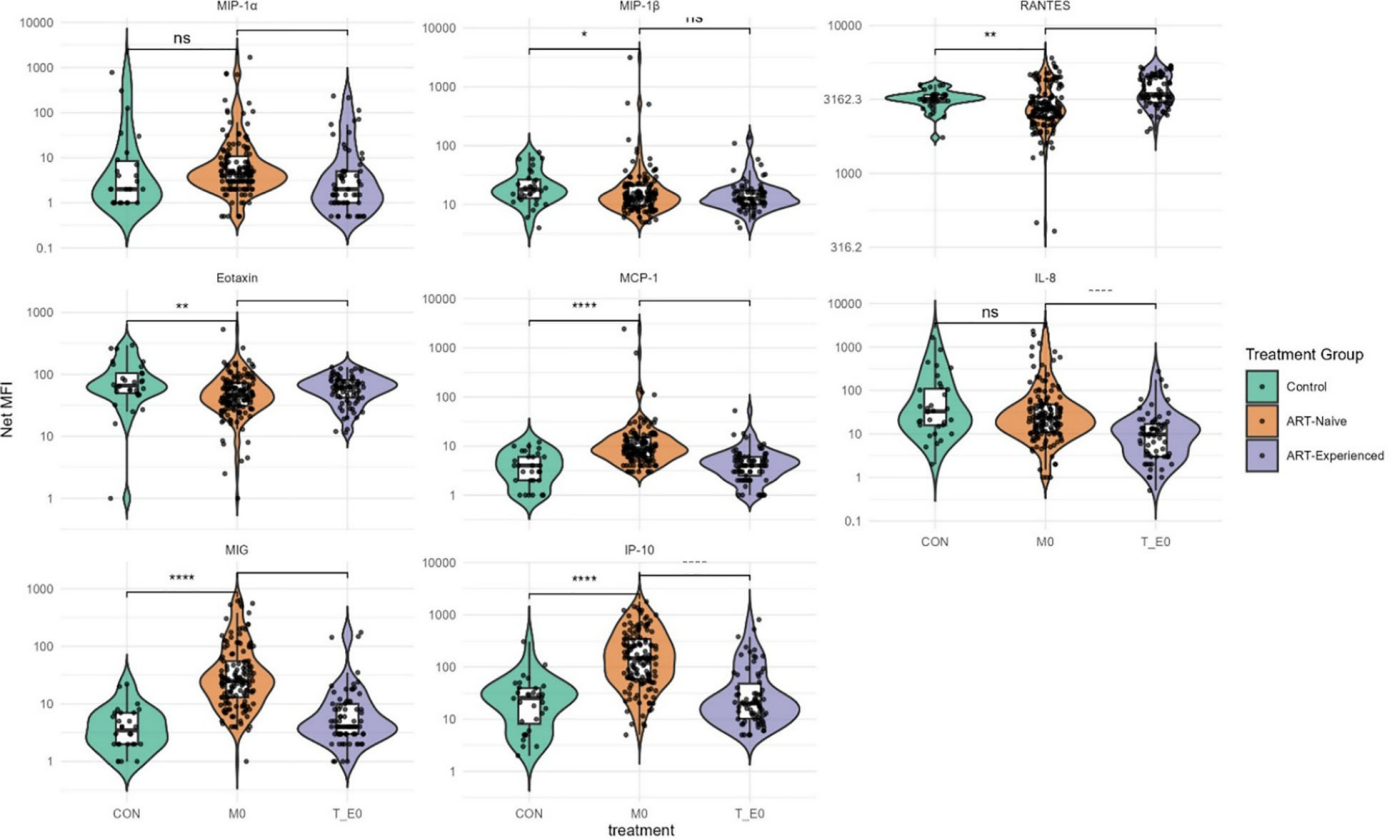
Chemokine levels in ART-naïve, ART-experienced, and control individuals. Violin plots show distributions of MIP-1α, MIP-1β, RANTES, Eotaxin, MCP-1, IL-8, MIG, and IP-10 across study groups. Data are expressed as Net MFI (log scale). Horizontal lines represent medians with 25th and 75th percentiles. Statistical comparisons were performed using nonparametric tests, with significance thresholds denoted as ****p < 0.0001; ***p < 0.001; **p < 0.01; *p < 0.05; ns, not significant.

ART-naïve participants had significantly elevated levels of several pro-inflammatory cytokines, particularly IL-6 and IL-12/IL-23p40, whereas IL-1β was significantly reduced compared to the control group (**Figure 1**). Additionally, cytokines involved in T-cell homeostasis and activation, including IL-15, IL-2, IL-2R, and IL-7, were significantly higher in ART-naïve individuals than in uninfected controls (**Figure 1**). In contrast, levels of the anti-inflammatory cytokine IL-1Ra were significantly lower in ART-naïve individuals (**Figure 2**). Chemotactic cytokines such as MCP-1, IP-10, and MIG were also significantly elevated in ART-naïve individuals compared to controls (**Figure 3**).

Following ART initiation, cytokine and chemokine profiles shifted markedly. In ART-experienced individuals, there was a significant reduction in pro-inflammatory cytokines including GM-CSF, IL-12/IL-23p40, IL-6, IL-15, IL-17, and TNF-α compared to ART-naïve participants (**Figure 1**). Notably, anti-inflammatory cytokines IL-10 and IL-1Ra were also significantly reduced in ART-experienced individuals compare to ART-naïve (**Figure 2**). Among chemokines, Eotaxin and RANTES were significantly increased in ART-experienced whereas MCP-1, IL-8, MIG, and IP-10 were significantly decreased compared to ART-naïve (**Figure 3**).

### Cytokines and chemokines as biomarkers for HIV progression

A Cox proportional hazards regression was used to access the predictive capacity of cytokines andchemokines for HIV disease progression among ART-naive, six and twelve months follow up participants (**Supplementary Figure 1**). The model demonstrated strong predictive performance (AIC = 679.94, concordance index = 0.84) with a highly significant global log-rank p-value (p = 7.07 × 10^-5^). Notably, lower levels of IL-1β (HR = 0.111, 95% CI: 0.0231–0.54, p = 0.006) and IL-10 (HR = 0.176, 95% CI: 0.0551–0.56, p = 0.003) were significantly associated with a higher likelihood of achieving virologic control. In contrast, elevated levels of GM-CSF (HR = 2.992, 95% CI: 1.118–8.01, p = 0.029) were associated with uncontrolled viraemia.

### Modelling cytokines and chemokines as predictors of viral load

**Figure 4:**
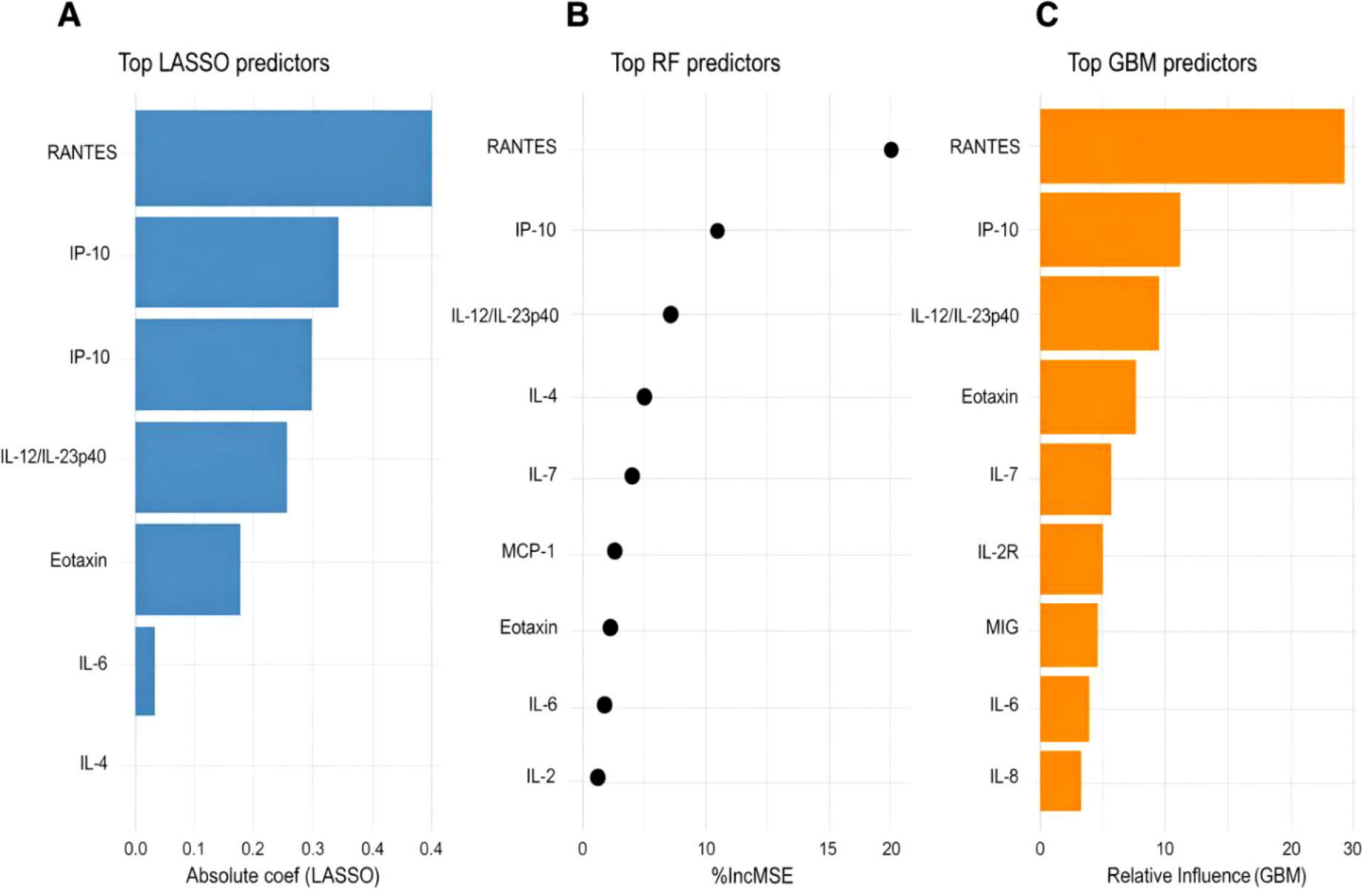
Top cytokine predictors identified by three models. Variable importance plots for **(A)** LASSO regression (absolute standardized coefficients), **(B)** Random Forest (% increase in mean squared error upon permutation), and **(C)** GBM (relative influence).

To evaluate cytokine predictors of HIV viral load, we applied LASSO regression, Random Forest (RF), and Gradient Boosting Machine (GBM) models. All three models demonstrated moderate predictive performance, with predicted versus observed log viral load showing good calibration (**Supplementary Figure 2**). RANTES and IP-10 were the strongest predictors, followed by IL-12/IL-23p40, Eotaxin, and IL-7 (**Figure 4**). Partial dependence analyses further highlighted non-linear cytokine–viral load relationships. RANTES exhibited an inverse association with viral load, while IP-10 and IL-12/IL-23p40 displayed positive effects, and Eotaxin and IL-7 showed threshold-dependent influences (**Supplementary Figure 3**).

### Cytokine and chemokine network in HIV pathogenesis

**Figure 5:**
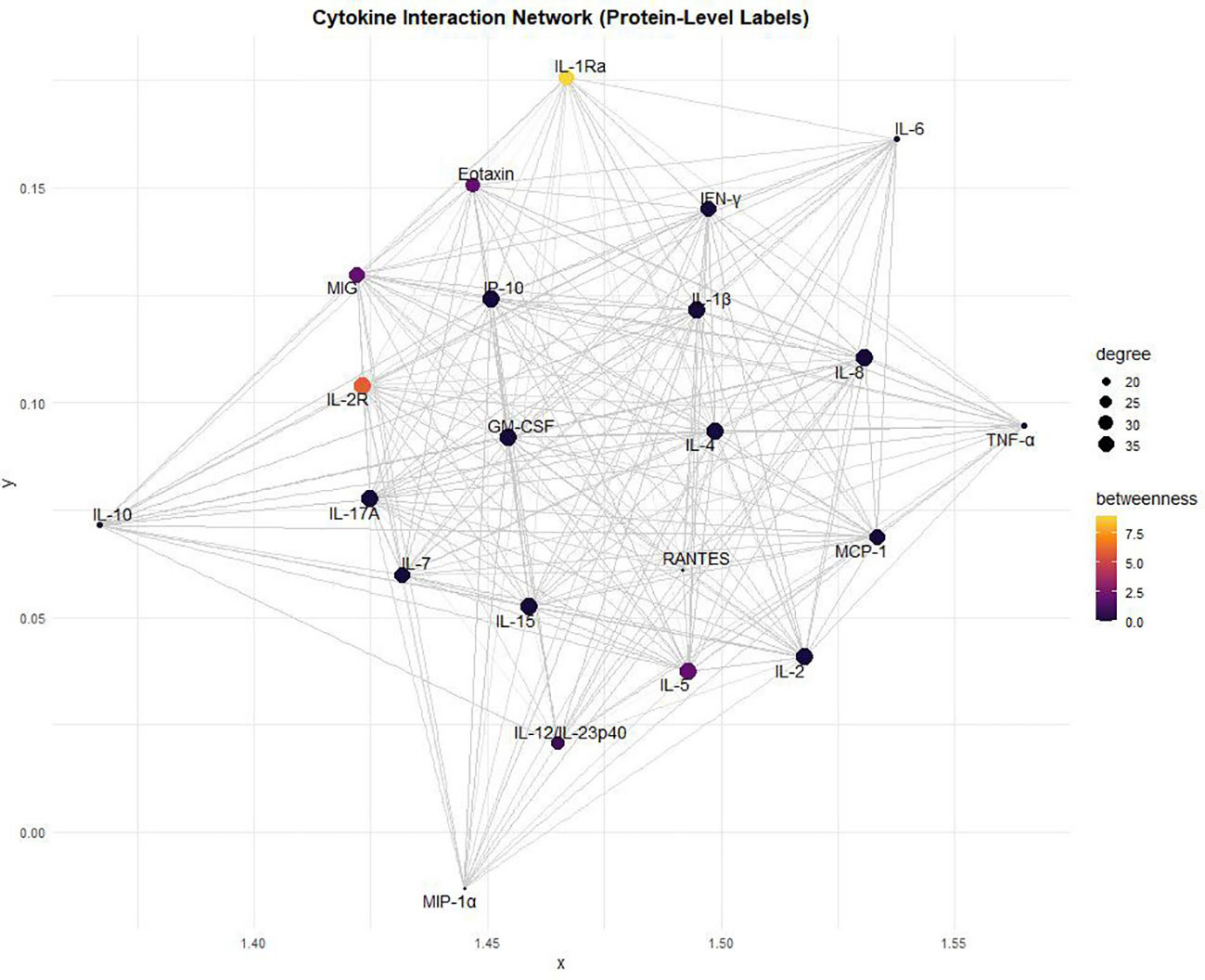
Cytokine interaction network for HIV pathogenesis. Nodes represent cytokines, with size proportional to degree (number of connections) and color gradient indicating betweenness centrality. Edges denote cytokine–cytokine interactions, highlighting key bridging cytokines within the network.

The cytokine interaction network in HIV pathogenesis revealed a complex web of interactions between key pro-inflammatory and immunoregulatory cytokines (**Figure 5**). IL-1Ra emerged as a central regulatory node, exhibiting the highest betweenness centrality. Other highly connected cytokines included TNF-α, IL-6, IL-17, and IL-10. Chemokines such as RANTES, MIG, and IP-10 formed strongly interconnected nodes, with Eotaxin also showing notable centrality. IFN-γ, IL-2, IL-7, GM-CSF, IL-1β, and MIP-1α were also integrated into the network (**Figure 5**).

### IP-10 and RANTES correlate with viral load

**Figure 6:**
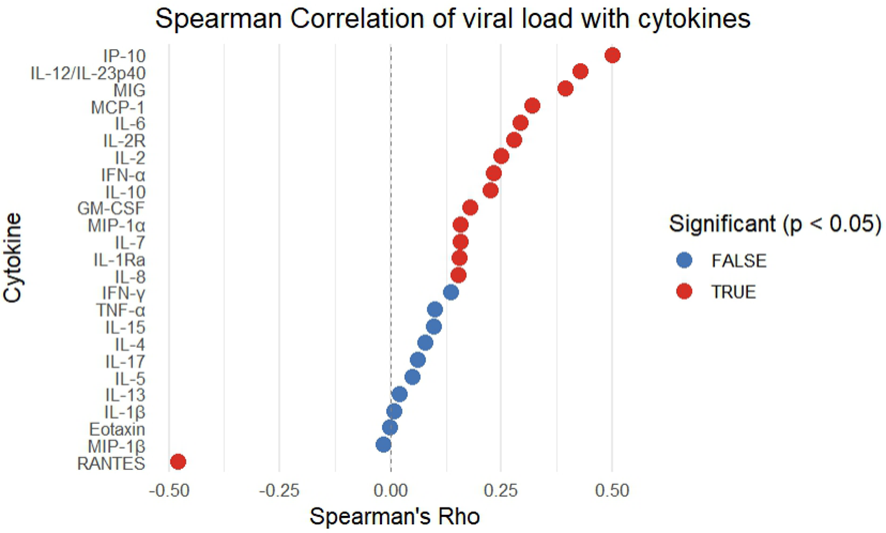
Correlation of cytokine and chemokines levels with HIV viral load in ART-naïve and ART-experienced individuals. Spearman correlation analysis was performed to assess the association between plasma cytokine concentrations and viral load across all HIV-infected participants. Cytokines showing significant correlations (p < 0.05) are highlighted in red, whereas non-significant correlations (p ≥ 0.05) are shown in blue. Positive Spearman’s rho values indicate direct associations between cytokine expression and viral load, while negative values reflect inverse relationships.

Spearman correlation analysis identified IP-10 as the strongest positive correlate of viral load, while RANTES showed a significant negative correlation (ρ ≈ –0.45) (**Figure 6**). Additional positive correlations with viral load were observed for IL-12/IL-23p40, MIG, MCP-1, IL-6, IL-2R, IL-2, and IFN-α.

### Gene ontology enrichment analysis

**Figure 7:**
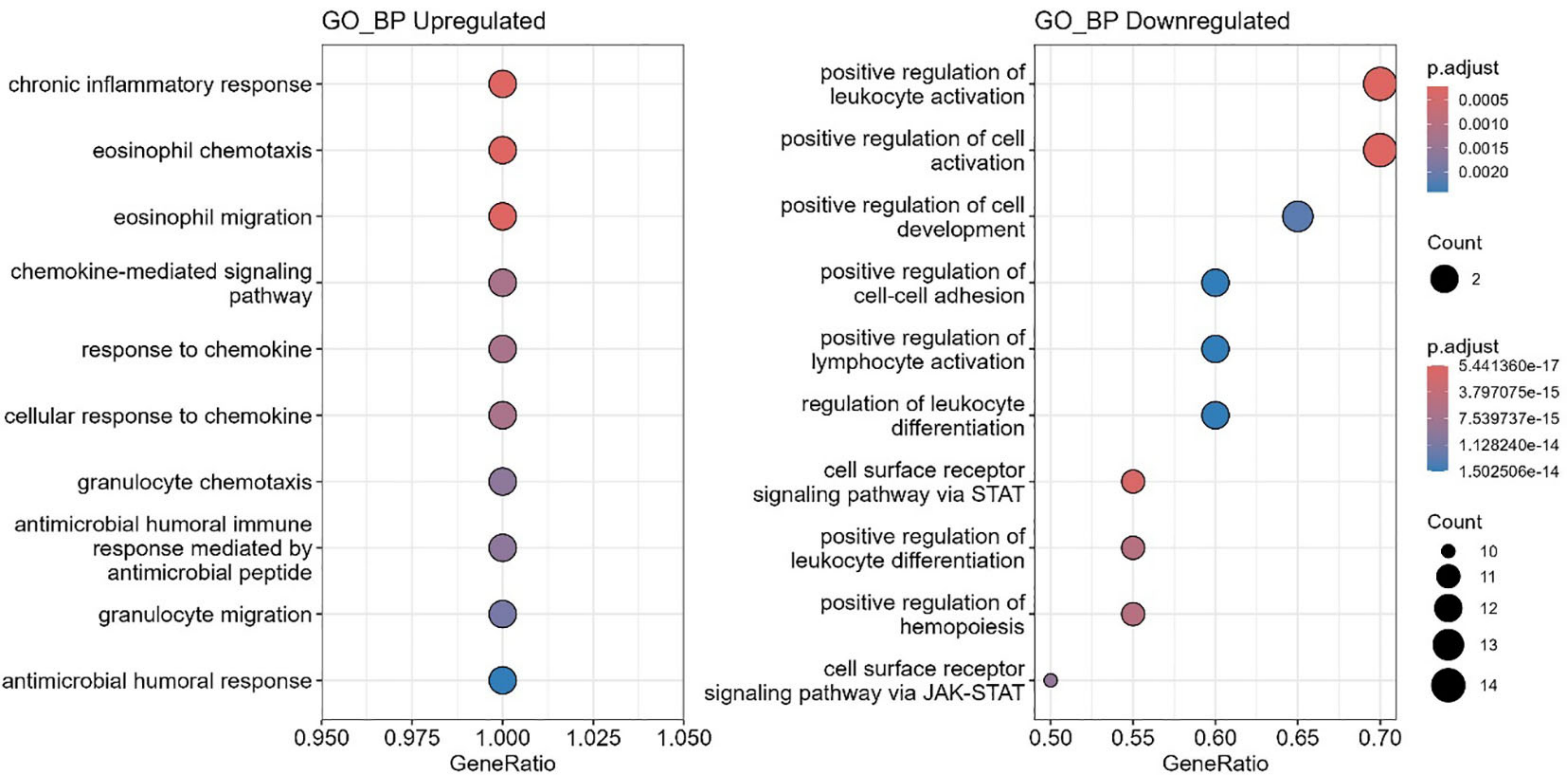
Enriched biological functions. Gene Ontology (GO) enrichment analysis of biological processes (GO_BP) was performed using *clusterProfiler*. Upregulated genes were significantly enriched in inflammatory and chemotactic processes (left panel), whereas downregulated genes were primarily associated with immune regulation, including leukocyte activation and JAK-STAT signaling pathways (right panel). Dot size corresponds to the number of genes in each term, and dot color represents the adjusted p-value.

**Figure 8:**
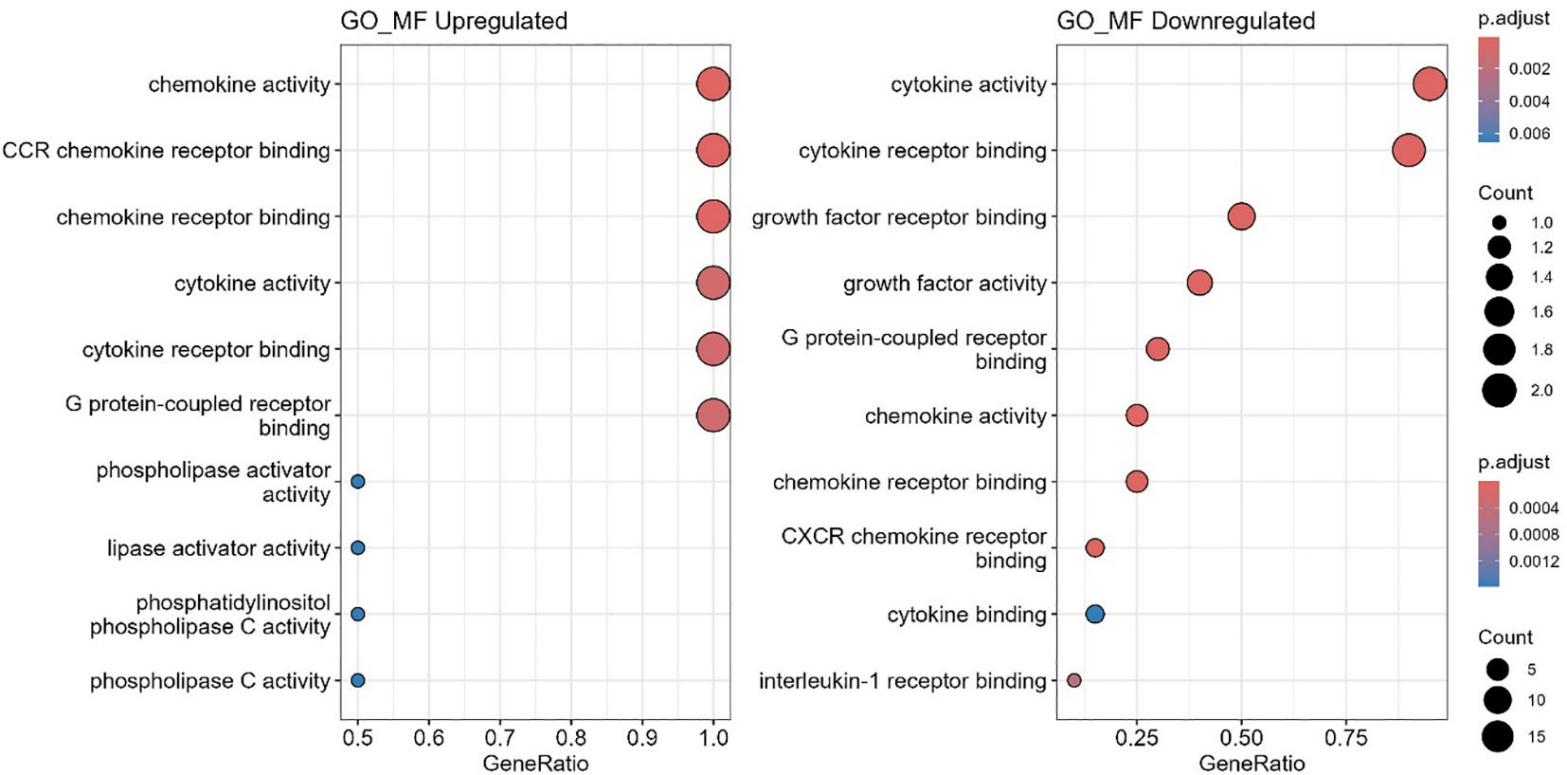
Enriched gene ontology (GO) molecular functions. Bubble plots show significantly enriched GO molecular function (GO_MF) terms for differentially expressed genes. Upregulated terms are shown on the left, and downregulated terms on the right. GeneRatio represents the proportion of genes associated with each term; bubble size indicates gene count, and color reflects adjusted p-values (p.adjust).

Gene ontology (GO) enrichment analysis revealed distinct immune processes altered between ART-naïve and ART-experienced groups (**Figures 7**, **8**; **Supplementary Figure 4**). Upregulated biological processes included chronic inflammatory response, eosinophil migration, chemokine-mediated signaling, granulocyte chemotaxis, and antimicrobial humoral responses. In contrast, pathways related to leukocyte activation, differentiation, and lymphocyte activation were downregulated.

At the molecular function level, chemokine activity, cytokine receptor binding, and G protein–coupled receptor binding were enriched, along with phospholipase activator activity. Downregulated functions included cytokine activity, growth factor receptor binding, and CXCR chemokine receptor interactions. In the cellular component category, downregulated pathways were mainly associated with the external plasma membrane and receptor complexes.

### KEGG pathway enrichment analysis

**Figure 9:**
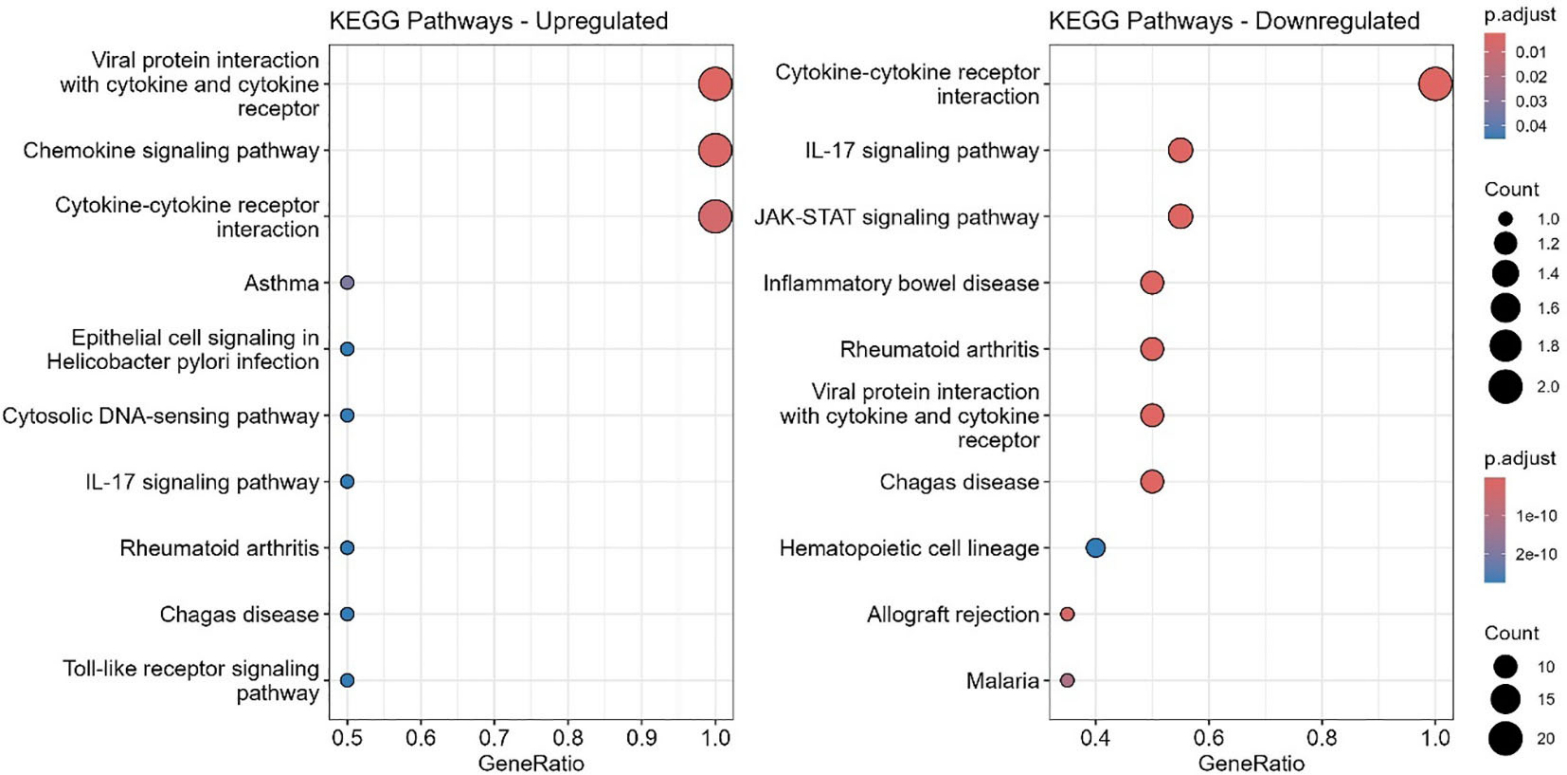
KEGG pathway enrichment analysis of top 10 differentially expressed genes. Bubble plots show significantly enriched KEGG pathways for upregulated (left) and downregulated (right) genes. GeneRatio represents the proportion of genes associated with each pathway. Dot size corresponds to the number of genes, and dot color reflects adjusted p-values (p.adjust), with darker red indicating higher significance. Upregulated genes are enriched in pathways related to viral protein interaction, chemokine signaling, and cytokine-cytokine receptor interaction, whereas downregulated genes are associated with cytokine signaling, IL-17, JAK-STAT pathways, and immune-related diseases.

KEGG pathway enrichment analysis of cytokines and chemokines that differed significantly between ART-naïve and ART-experienced groups revealed enrichment of immune pathways, including viral protein–cytokine interactions, chemokine signaling, and cytokine–cytokine receptor interactions (**Figure 9**). Downregulated pathways included IL-17 and JAK-STAT signaling, along with those related to inflammatory bowel disease, rheumatoid arthritis, malaria, Chagas disease, hematopoietic lineage differentiation, and allograft rejection.

## Discussion

Cytokine and chemokine variations across the study groups highlight the dual roles of immune activation and regulation in HIV pathogenesis, disease progression, and ART response. In ART-naïve individuals, elevated pro-inflammatory cytokines, particularly IL-6 and IL-12/IL-23p40, reflected a state of chronic immune activation, a hallmark of HIV pathogenesis (27). Persistent inflammation drives viral replication, CD4+ T cell depletion, and accelerated disease progression (28). Similarly, higher levels of IL-15, IL-2, IL-2R, and IL-7, suggest attempts at immune reconstitution, even in the absence of ART. This sustained activation promotes T-cell exhaustion and dysregulation (29).

Reduced IL-1β in ART-naïve individuals suggests impaired innate immune signaling, which weakens early antiviral responses and facilitates viral persistence (30). Likewise, the decreased IL-1Ra, a key anti-inflammatory cytokine, points to a limited capacity to counterbalance inflammation, further fueling immune activation, exhaustion and disease progression (31). Elevated chemotactic cytokines, including MCP-1, IP-10, and MIG, likely enhance recruitment of activated immune cells to sites of infection, providing more target cells for HIV replication and enhancing viral dissemination (28). This contributes to systemic inflammation, ultimately enhancing the establishment and maintenance of viral reservoirs.

ART initiation resulted in significantly reduced pro-inflammatory cytokines, including GM-CSF, IL-6, IL-12/IL-23p40, IL-1β, and TNF-α. This indicates a restoration of regulatory balance by the suppression of immune hyperactivation as similarly reported in other cohorts (32). As treatment reduces circulating virus, immune stimulation diminishes, leading to a downstream reduction in multiple inflammatory and chemotactic pathways. Reduced IL-15 and other Th1 cytokines (IFN-γ, IFN-α, IL-2, IL-7, IL-15, IL-2R) highlight downregulation of immune activation, critical for preserving long-term immune competence. Importantly, lower IL-10 and IL-1Ra in ART-experienced individuals likely reflect diminished need for compensatory immunosuppression following viral suppression (33). Conversely, chemokines with HIV entry-blocking properties, such as Eotaxin and RANTES, were significantly elevated post-ART, consistent with protective roles against viral re-entry (32). Reduced chemotactic cytokines (MCP-1, IL-8, MIG, and IP-10) indicates decreased immune cell trafficking and inflammation, contributing to overall immune stabilization.

Of note, ART-experienced individuals showed reduced IL-17, a Th17 cytokine essential for maintaining mucosal immunity, particularly in the gastrointestinal tract. This suggests incomplete restoration of gut-associated lymphoid tissue (GALT), consistent with prior reports (34, 35). Such impairment may perpetuate microbial translocation and chronic inflammation despite systemic viral suppression.

Specific cytokine associations were also observed. Lower IL-1β and IL-10 were associated with virologic control, suggesting that reduced expression supports a less inflammatory milieu favorable for viral suppression (36). Elevated GM-CSF was linked to unsuppressed viral load, consistent with its role in driving myeloid activation and inflammatory responses that can promote viral replication and reservoir maintenance (37, 38). These findings highlight the prognostic potential of IL-1β, IL-10, and GM-CSF for stratifying patients prior to ART initiation.

Viral load correlations confirmed IP-10 as most strongly associated with viraemia, reinforcing its role in systemic inflammation and replication (39). IL-2R, IL-6, and MCP-1 also correlated positively, while RANTES displayed a negative correlation, consistent with its competitive blockade of CCR5-mediated HIV entry (40, 41). Similarly, in East Africa, the REALITY trial found elevated IL-6 and IP-10 to be associated with increased all-cause mortality, whereas higher IL-23, IL-2, and RANTES were associated with reduced mortality (42).

Complementary machine learning analyses further identified RANTES and IP-10 as the most consistent predictors of HIV viral load across three independent modelling approaches. RANTES (CCL5) predicted lower viral load, consistent with its role as a CCR5 ligand that restricts HIV entry. In contrast, IP-10 predicted higher viral load, in line with its role as a marker of immune activation and disease progression as previously reported (43). IP-10 has also been reported to correlate with increasing viral loads in Southern Africa (44). The importance of IL-12/IL-23p40, IL-7, and IL-2R in the models additionally implicates dysregulated T-cell homeostasis and pro-inflammatory signaling in viral persistence. Notably, non-linear models (RF, GBM) captured threshold and saturation effects missed by LASSO, underscoring the value of machine learning in unravelling complex immune–viral dynamics.

Cytokine network analysis provides important insights into the immune signaling dynamics underlying HIV pathogenesis. IL-1Ra emerged as a central regulatory node with the highest betweenness centrality, consistent with its role in modulating immune responses and mitigating excessive inflammation (31). GM-CSF, TNF-α, IL-1β, and MIP-1α exhibited high degree centrality, highlighting their roles in sustaining chronic inflammation. GM-CSF promotes M1 macrophage activation, creating a pro-inflammatory environment that supports viral persistence (38, 45). Similarly, TNF-α and IL-1β are potent drivers of systemic inflammation and neurotoxicity, contributing to HIV-associated neurocognitive impairment and immune dysregulation (46). Chemokines such as MCP-1, MIG, and IP-10 occupied highly connected regions, consistent with their roles in recruiting CCR2^+^ and CXCR3^+^ immune cells to infection sites and exacerbating viral dissemination and chronic immune activation (39). Collectively, these findings suggest that cytokines with high network centrality are not merely bystanders but active drivers of HIV pathogenesis.

Functional enrichment analyses provided additional context. Gene Ontology (GO) revealed upregulation of chemotaxis-related processes, including granulocyte and eosinophil migration, supporting ongoing inflammation despite ART (47, 48). Conversely, pathways regulating leukocyte activation and differentiation, including JAK-STAT signaling, were downregulated (49, 50), suggesting impaired adaptive immune coordination. Molecular function analysis showed upregulation of chemokine receptor binding, cytokine activity, and G protein-coupled receptor (GPCR) signaling but downregulation of interleukin-1-receptor activity and CXCR binding, pointing to robust inflammatory signaling but reduced immune responsiveness (39, 51, 52). At the cellular component level, downregulation of plasma membrane receptor complexes suggests impaired immune recognition of infected cells (53).

KEGG pathway analysis confirmed enrichment of inflammatory pathways, including chemokine signaling and cytokine–receptor interactions (26). Downregulation of JAK-STAT, IL-17, and hematopoietic cell lineage pathways highlights persistent immune exhaustion and impaired hematopoiesis, consistent with previous reports (54–57).

## Conclusion

ART initiation reduces circulating virus, thereby reducing immune activation. Thus, there is a concomitant reduction in systemic inflammation and a partial restoration of immune function in PLWH. However, recovery remains incomplete and functionally dysregulated. Persistent chemotactic signaling sustains immune cell trafficking, while downregulation of activation and differentiation pathways limits antigen-specific responses. This creates a paradox of numerical immune recovery but functional compromise, contributing to viral non-suppression despite ART.

Key cytokines—IL-1β, IL-10, GM-CSF, RANTES, and IP-10 emerge as potential prognostic markers of disease progression. Targeted interventions could include restoring mucosal immunity through IL-17 modulation, reducing immune activation via IP-10 inhibition, and enhancing RANTES activity to block HIV entry. Together, these findings highlight cytokine signatures as critical determinants of HIV persistence and immune recovery and support their use in risk stratification and therapeutic development.

## Data Availability

The original contributions presented in the study are included in the article/**Supplementary Material**. Further inquiries can be directed to the corresponding author.

## Supplementary material

Supplementary Figure 1 Cytokine hazard ratios for HIV progression. Forest plot of hazard ratios (HRs) for individual cytokines derived from a Cox proportional hazards model. HRs >1 indicate an increased risk of viral non-suppression, whereas HRs <1 suggest virologic control. Horizontal lines represent 95% confidence intervals (CIs), and statistical significance is denoted by ***.

Supplementary Figure 2 Model performance (predicted vs. observed). Scatterplots of predicted versus observed log viral load (log_10_ copies/mL) for the test set using LASSO regression, Random Forest (RF), and Gradient Boosting Machine (GBM). Each point represents an individual sample. The fitted regression line (blue) with 95% confidence band (gray) indicates model calibration.

Supplementary Figure 3 Partial dependence of top cytokines. Partial dependence plots (PDPs) for the top 10 cytokines identified across models, shown separately for RF (blue) and GBM (orange). Each panel depicts the marginal effect of one cytokine on predicted log viral load, holding other variables constant. Non-linear associations and threshold effects are evident.

Supplementary Figure 4 Enriched cellular components. GO cellular component (GO_CC) enrichment analysis of downregulated genes shows enrichment in membrane-associated components, particularly the external side of the plasma membrane and the plasma membrane signalling receptor complex. Dot size indicates gene count, and color represents adjusted p-values.
