# Supplementary material for: JAK-STAT and IL-17 pathway dysregulation underlies persistent immune dysfunction in ART-experienced people living with HIV in Ghana
Source: Front Immunol. 2026 Feb 10;17:1753475. doi: 10.3389/fimmu.2026.1753475 (PMC12929546; doi:10.3389/fimmu.2026.1753475)
Supplement: Supplementary Figure 1 — Cytokine hazard ratios for HIV progression. Forest plot of hazard ratios (HRs) for individual cytokines derived from a Cox proportional hazards model. HRs >1 indicate an increased risk of viral non-suppression, whereas HRs <1 suggest virologic control. Horizontal lines represent 95% confidence intervals (CIs), and statistical significance is denoted by ***. [file DataSheet1.pdf]

Supplementary Data

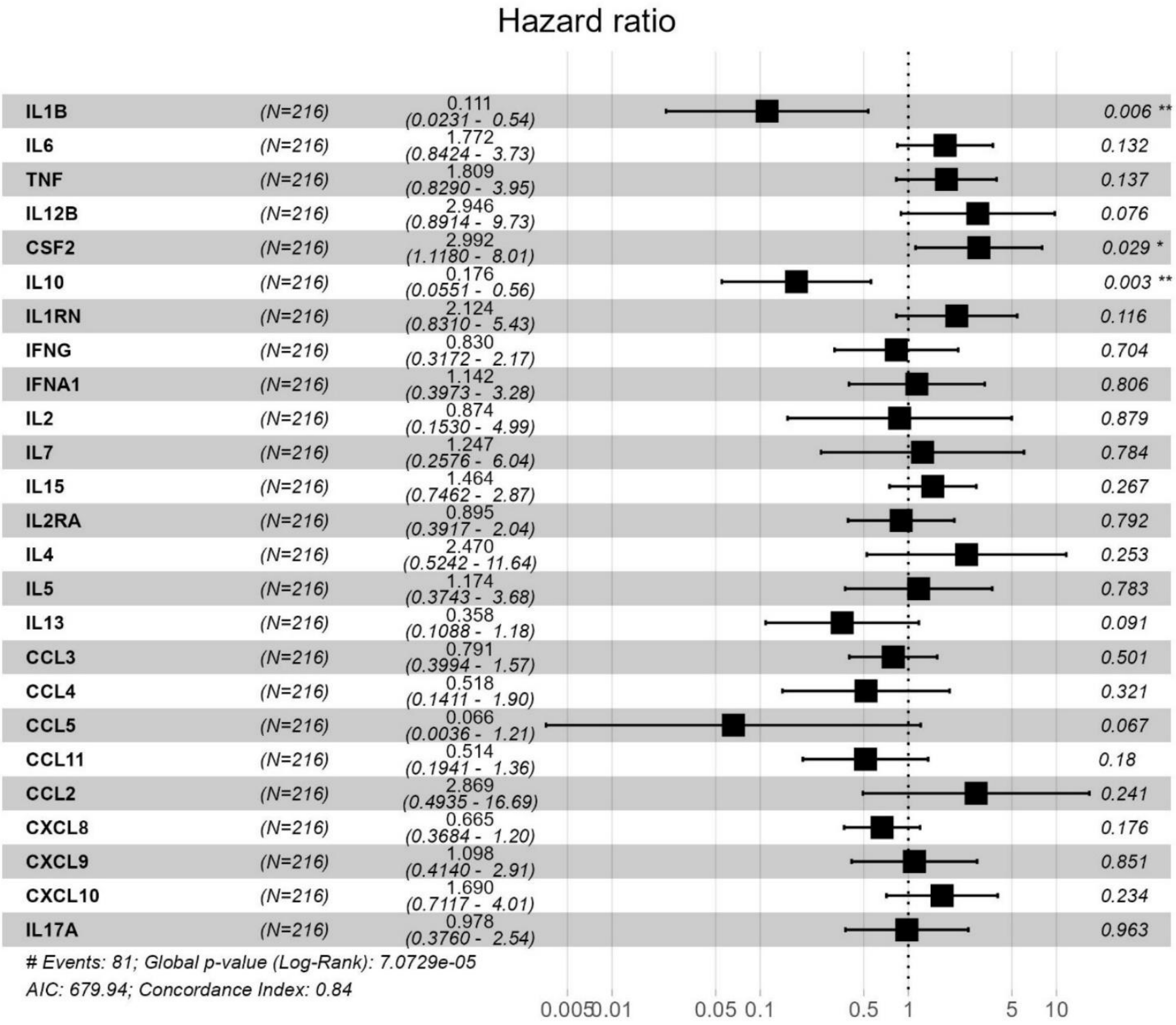

Supplementary figure 1. Cytokine hazard ratios for HIV progression. Forest plot of hazard ratios (HRs) for individual cytokines derived from a Cox proportional hazards model. HRs >1 indicate an increased risk of viral non-suppression, whereas HRs <1 suggest virologic control. Horizontal lines represent 95% confidence intervals (CIs), and statistical significance is denoted by ***.

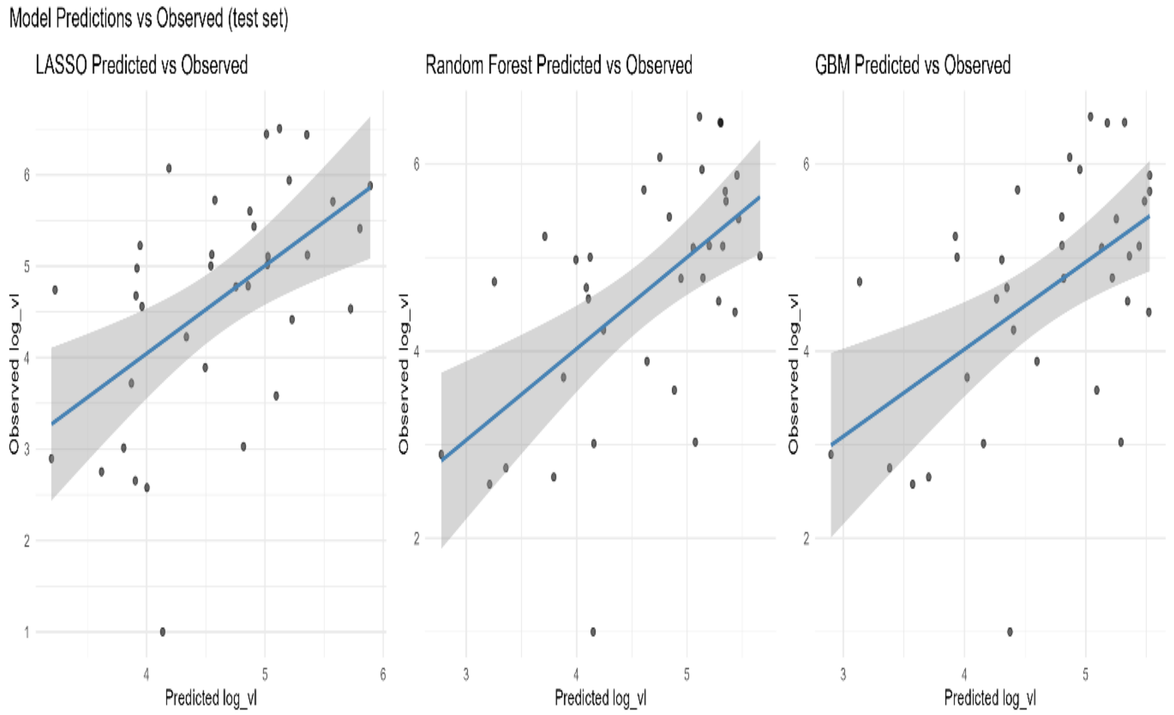

Supplementary figure 2. Model performance (Predicted vs. Observed). Scatterplots of predicted versus observed log viral load (log_{10} copies/mL) for the test set using LASSO regression, Random Forest (RF), and Gradient Boosting Machine (GBM). Each point represents an individual sample. The fitted regression line (blue) with 95% confidence band (gray) indicates model calibration.

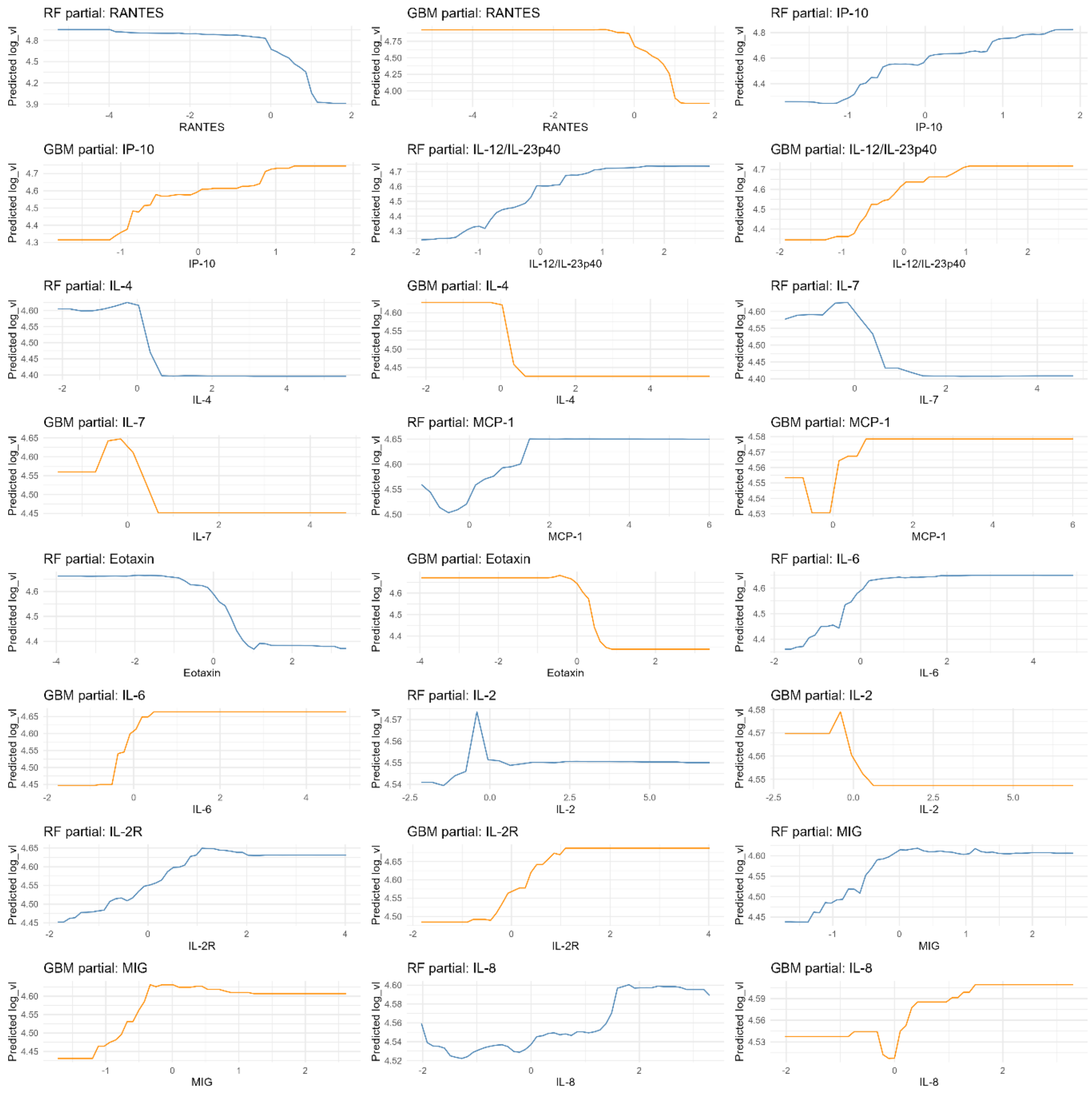

Supplementary figure 3. Partial dependence of top cytokines. Partial dependence plots (PDPs) for the top 10 cytokines identified across models, shown separately for RF (blue) and GBM (orange). Each panel depicts the marginal effect of one cytokine on predicted log viral load, holding other variables constant. Non-linear associations and threshold effects are evident.

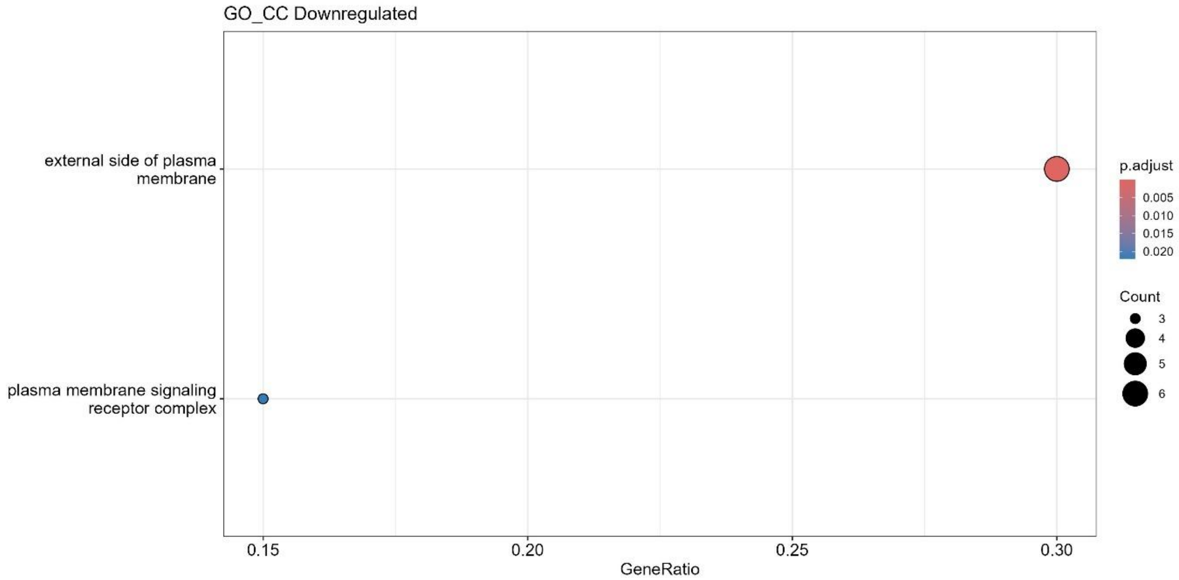

Supplementary figure 4**. Enriched Cellular Components.** GO cellular component (GO_CC) enrichment analysis of downregulated genes shows enrichment in membrane-associated components, particularly the external side of the plasma membrane and the plasma membrane signalling receptor complex. Dot size indicates gene count, and colour represents adjusted p-values.
